# Hemodynamics in a giant intracranial aneurysm characterized by *in vitro* 4D flow MRI

**DOI:** 10.1371/journal.pone.0188323

**Published:** 2018-01-04

**Authors:** Omid Amili, Daniele Schiavazzi, Sean Moen, Bharathi Jagadeesan, Pierre-François Van de Moortele, Filippo Coletti

**Affiliations:** 1 Department of Aerospace Engineering and Mechanics, University of Minnesota, Minneapolis, MN, United States of America; 2 Department of Applied and Computational Mathematics and Statistics, University of Notre Dame, Notre Dame, IN, United States of America; 3 Department of Radiology, University of Minnesota, Minneapolis, MN, United States of America; 4 Department of Neurology, University of Minnesota, Minneapolis, MN, United States of America; 5 Department of Neurosurgery, University of Minnesota, Minneapolis, MN, United States of America; 6 Center for Magnetic Resonance Research, University of Minnesota, Minneapolis, MN, United States of America; 7 St. Anthony Falls Laboratory, University of Minnesota, Minneapolis, MN, United States of America; Worcester Polytechnic Institute, UNITED STATES

## Abstract

Experimental and computational data suggest that hemodynamics play a critical role in the development, growth, and rupture of cerebral aneurysms. The flow structure, especially in aneurysms with a large sac, is highly complex and three-dimensional. Therefore, volumetric and time-resolved measurements of the flow properties are crucial to fully characterize the hemodynamics. In this study, phase-contrast Magnetic Resonance Imaging is used to assess the fluid dynamics inside a 3D-printed replica of a giant intracranial aneurysm, whose hemodynamics was previously simulated by multiple research groups. The physiological inflow waveform is imposed in a flow circuit with realistic cardiovascular impedance. Measurements are acquired with sub-millimeter spatial resolution for 16 time steps over a cardiac cycle, allowing for the detailed reconstruction of the flow evolution. Moreover, the three-dimensional and time-resolved pressure distribution is calculated from the velocity field by integrating the fluid dynamics equations, and is validated against differential pressure measurements using precision transducers. The flow structure is characterized by vortical motions that persist within the aneurysm sac for most of the cardiac cycle. All the main flow statistics including velocity, vorticity, pressure, and wall shear stress suggest that the flow pattern is dictated by the aneurysm morphology and is largely independent of the pulsatility of the inflow, at least for the flow regimes investigated here. Comparisons are carried out with previous computational simulations that used the same geometry and inflow conditions, both in terms of cycle-averaged and systolic quantities.

## 1 Introduction

Brain aneurysms have a prevalence rate of 3-5% in the general population [1, 2] having an overall rupture risk of 1.2% within 5 years from diagnosis [3]. According to the Brain Aneurysm Foundation [4], there are more than three brain aneurysm ruptures every hour across the United States and approximately half a million deaths worldwide each year.

The formation and growth of an aneurysm is the result of a complex interaction of multiple factors. In addition to genetic, physiological, environmental factors, and tissue mechanics, it is believed that fluid dynamics play an important role in the formation and development of arterial aneurysms at certain sites, e.g. see [5, 6]. The majority of the brain aneurysms are observed at bifurcations or arterial branches located in the circle of Willis where the flow topology is complex. At these locations, the aneurysm geometrical shape parameters have been suggested to correlate with the progression of the lesion with metrics such as aneurysm size, aspect ratio, size ratio, undulation index, non-sphericity index, and ellipticity index, see for example [7]. Of these, the impact of the aneurysm size on the rupture risk remains the most widely studied metric.

Intracranial aneurysms are normally considered small if their largest dimension is smaller than ∼10 mm, large if the corresponding measurement is in the range of 10-25 mm, and giant if larger than 25 mm. The risk of subarachnoid haemorrhage is known to increase with an increase in the size of the aneurysm [3, 8, 9]. For example, a 30 mm aneurysm at the tip of the basilar artery has a rupture risk of 50-60% within 5 follow-up years [9]. Location of the aneurysm in the posterior circulation is also known to increase the risk of rupture. However, both size and location of the aneurysm remain sub-optimal predictors of rupture risk as evidenced by the fact that a significant number of ruptured cerebral aneurysms measure less than 7 mm [10, 11].

Therefore, in addition to the observation of the simple morphological aspects mentioned above, in recent years there has been an increasing interest in the image-based fluid dynamics assessment of brain aneurysms due to availability of high resolution CT/MRI scans. Elevated or abnormally low wall shear stress (WSS) or pressure, oscillatory shear index (OSI), relative residence time (RRT), energy loss (EL) across the aneurysm, and Aneurysm number (*An*) are among the key factors that have been studied to understand the flow dynamics in aneurysms with the aim of quantitatively characterizing the risk for aneurysm growth and rupture. For example, in the computational fluid dynamics (CFD) study by [12], the Aneurysm number (the ratio of the transport time scale to vortex formation time scale) was studied as a predictor for rupture risk. It was argued that for *An* <1, a stable shear layer is attached to inflow and outflow walls and a cavity type flow is expected. However, for *An* >1, the shear layer is separated from the outflow wall, and the unsteadiness of the unstable shear layer leads to a vortex ring formation at the neck/artery interface (for the relative position of the body, dome, and neck of different brain aneurysms see [6]). It was further argued that aneurysms under the vortex ring mode are likely to grow and rupture. The clinical study on carotid artery aneurysms by [13] also observed an increased pulsatility index within the aneurysm for ruptured cases implying higher *An*. A persistent vortex ring structure in an elastase-induced sidewall aneurysm under *An* >1 was also observed by [14].

As evidenced by the above studies, most of our current understanding of the role of the metrics in brain aneurysms comes from computational fluid dynamics with comparably few experimental studies to support and validate the numerical simulations. For example, in the ASME 2012 Summer Bioengineering Conference CFD Challenge (for brevity, 2012 CFD Challenge), 27 groups simulated the Newtonian pulsatile flow in a giant brain aneurysm with rigid walls. The only experimental validation available was the pressure drop across the entire aneurysm. Although the majority of groups were consistent in predicting the systolic pressure, there was much larger variability in the predicted systolic velocity pattern [15]. In this comparative study, identified outlier simulations based on the pressure map were not necessarily outliers based on the velocity map and vice versa. In addition, agreement in the systolic pressure/flow pattern did not necessarily imply a corresponding agreement in the mean values over the cardiac cycle. This motivates the need for rigorous *in vitro* validation of hemodynamic metrics at conditions matching the numerical simulations. Without detailed experimental studies supporting the computations, our understanding of aneurysm hemodynamics remains incomplete.

On the other hand, most previous experimental studies using *in vitro* replicas of intracranial aneurysms have used laser-based flow diagnostics such as point-wise laser doppler anemometry (LDA) or planar particle image/tracking velocimetry (PIV/PTV). A three-component three-dimensional (3C-3D) velocity field was reconstructed by [16] using stereoscopic PIV by stacking several non-simultaneously recorded planes. Le et al. (2013) [14] also successfully measured the full 3C-3D velocity field in an animal brain aneurysm using 3D particle tracking based on defocusing PIV. Nevertheless, the majority of experimental attempts to measure this three-dimensional and unsteady flow is limited to slices of the flow in the aneurysm sac. For example, see [17, 18, 19, 20, 21, 22, 23].

In the present study, we leverage magnetic resonance imaging (MRI) to perform 4D (i.e. three-dimensional and time-resolved) flow measurements in a giant brain aneurysm, aiming at investigating the volumetric velocity and pressure fields under a physiological cardiac waveform. In addition, this study provides experimental information for the same geometry and boundary condition that was previously analyzed by several groups in [15] and demonstrates the opportunities offered by 4D flow MRI measurements to investigate complex hemodynamics. The paper provides details of the experimental methodology including the fabrication of the aneurysm phantom, flow circuit design, 4D flow MRI measurements, pressure reconstruction based on the velocity field, pressure drop measurements, and details of data pre- and post-processing. Results are presented and discussed in detail and are briefly compared to the 2012 CFD Challenge data, followed by concluding remarks.

## 2 Methodology & experimental setup

### 2.1 Aneurysm model

The selected aneurysm geometry was first investigated by [24] and later was used in the 2012 CFD Challenge [15]. It is a giant pre-treatment aneurysm at the left internal carotid artery (ICA) with a stenosis proximal to the aneurysm orifice. The aneurysm ruptured 4 days following a flow-diverting stent treatment. The inlet diameter of the vessel is approximately 5.6 mm, and the maximum dimension in the aneurysm sac is approximately 26 mm. Although the spatial resolution in the flow measurements is high (see Section 2.3), to further increase the dynamic range, the original geometry has been scaled up by a factor of 2.0. The model is manufactured by 3D printing with a wall thickness of 3 mm at the W. M. Keck Center, University of Texas at El Paso. High resolution stereo-lithography with 25 *μ*m layers of Somos^®^ WaterShed XC 11122 guarantees hydro-dynamically smooth walls. The phantom reconstructed through MRI closely matches the 3D printing geometry confirming sub-millimeter precision.

**Fig 1:**
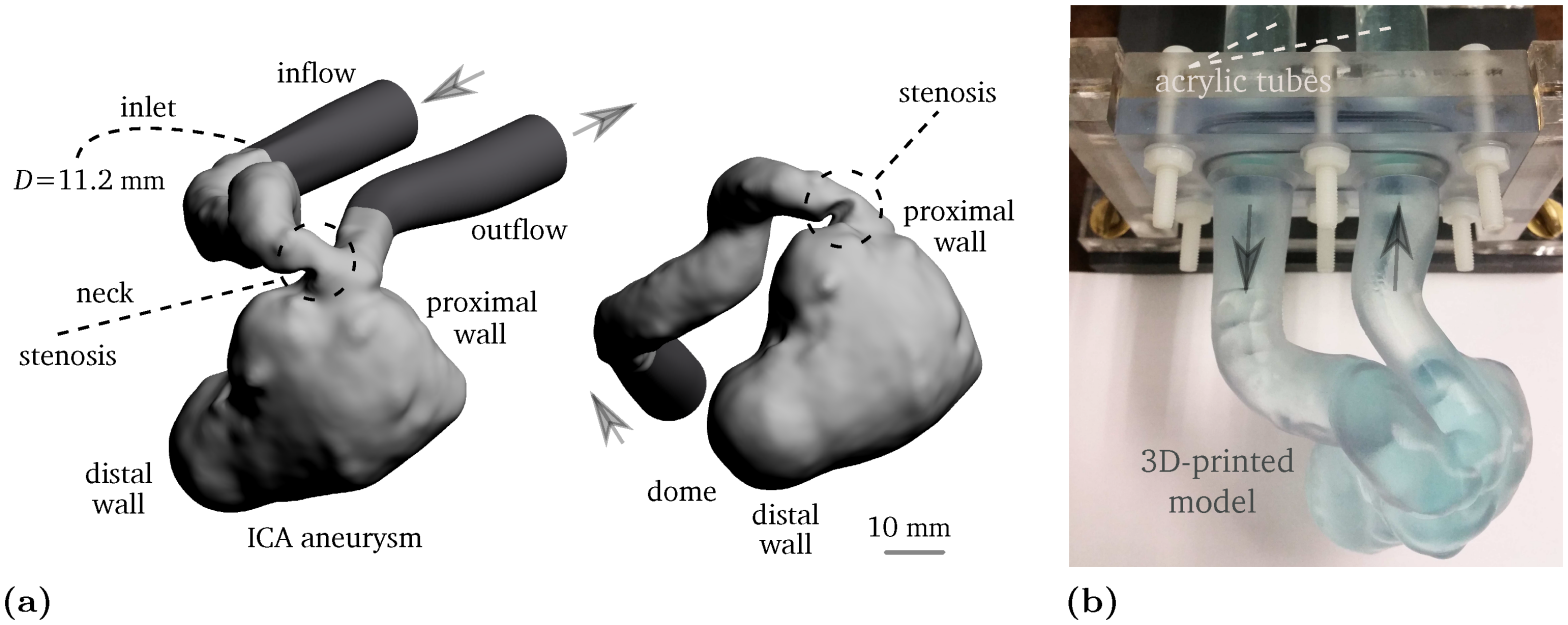
(a) Lumen of the giant pre-treatment internal carotid artery aneurysm in 2:1 scale. The light gray geometry indicates the STereoLithography file taken from [24], and the dark gray regions are the extensions that connect the lumen to the acrylic tubes. (b) 3D-printed phantom in 2:1 scale filled with water-glycerol mixture infused with CuSO_4_. The arrows represent the flow direction.

In order to establish well-defined inflow/outflow conditions, the inlet and outlet consist of acrylic tubes with an inner diameter of *D*_*t*_ = 12.7 mm and a length of *L*_*t*_ = 22*D*_*t*_. This allows for sufficient spacing between the lumen inlet/outlet and the corresponding connections to the flow circuit. Both pulsatile and steady state conditions are considered here. The steady flow configurations are used for comparison, to illustrate the influence of the oscillatory inflow. This is important, as it will be shown, the main hemodynamics aspects are similar under both steady and pulsatile inflow conditions. The inflow velocity profile at the inlet follows the Womersley solution [25] in case of the pulsatile flow measurement and it closely approximates the Poiseuille solution in the steady state flow scenario. The CAD and real geometries are shown in Fig 1(a) and 1(b), respectively. Throughout this study, the “inlet” as shown in Fig 1(a) refers to the inflow parent artery leading to the aneurysm sac with an inner diameter of *D* ≈ 11.2 mm.

### 2.2 Flow circuit

**Fig 2:**
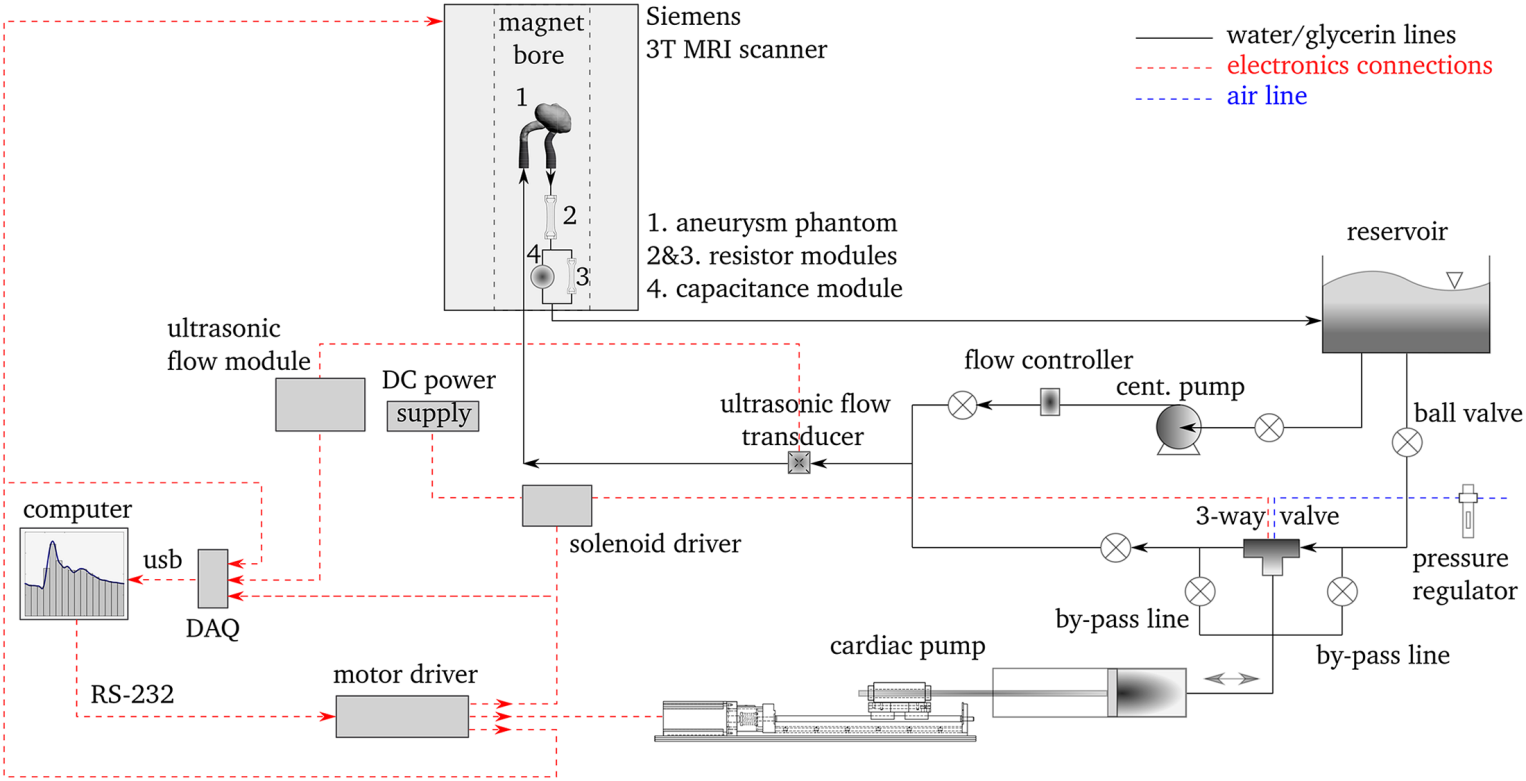
A schematic of the experimental setup. The black solid lines indicate the flow circuit, whereas the red dashed lines indicate the electronics connections. Note that the components are not to scale.

In order to generate the required flow, we use the flow loop schematically illustrated in Fig 2. To produce physiologically realistic flow waveforms, an in-house built cardiac pump consisting of a precision 2-phase hybrid stepper motor, a linear traverse stage, and a Teflon piston sliding in an acrylic cylinder is used. For switching between discharging and charging lines, a solenoid actuated Swagelok 3-way ball valve with a short response time of ∼0.2 s is used. This ensures the charging/discharging periods are isolated from each other and hence any reverse flow in the aneurysm model during the charging period is avoided.

A ME-6PXL Transonic flow transducer (0-5 LPM) is used to monitor the flow rate. It is an ultrasonic flow sensor with an absolute accuracy of ±10% of the reading, clamped onto a Tygon^®^ tube with an inner diameter of 6.35 mm and a wall thickness of 1.6 mm. A TS410 Transonic tubing flow module operated at a low-pass filter cutoff frequency of 40 Hz is used to obtain the high fidelity flow signal. Flow meter has been calibrated against timed collection of fluid. The signals for solenoid actuation, MRI gating, and flow rate are logged using a 14-bit National Instruments data acquisition system (USB-6001) at a sampling rate of 200 Hz.

A centrifugal pump is used to prime the flow loop and to drive the steady flow configuration. An Alicat Scientific flow controller (LCR-series 0-5 LPM) is used to remove any plausible upstream fluctuations due to the centrifugal pump. Reinforced plastic tubing with an inner diameter of 25.4 mm is used for all lines to ensure minimal compliance in the system while providing enough flexibility for connections. The impedance of the flow circuit mimics the arterial bed through standard Windkessel boundary conditions [26] and is controlled using two resistors made of capillary tubes and a module containing air mimicking the arterial capacitance.

A mixture of water with approximately 25% of glycerin by weight is used as the working fluid. CuSO_4_ with a concentration of ∼0.06 mol/L is added to the mixture. This does not change the fluid properties and enhances the MRI signal-to-noise ratio [27]. Measurements are acquired at a temperature of 20.5°C with a kinematic viscosity (*ν*) of approximately 2.0×10^−6^ m^2^/s and a density (*ρ*) equal to ∼1060 kg/m^3^ calculated based on the fluid’s refractive index [28]. The viscosity calculation is confirmed using a glass capillary viscometer. The use of the glycerin solution is advantageous compared to plain water as it allows us to use higher velocities in order to stay well above the noise floor of the PC-MRI measurements which in this case is less than 0.01 m/s. In addition, the input waveform period with the use of this glycerin solution is half that of plain water and therefore the MRI acquisition time is shorter.

The inflow waveform is taken from [24] (or “Pulsatile 2” case in [15]) and is scaled in flow rate (*Q*) and period (*T*) to match the desired Reynolds and Womersley numbers. The Reynolds number based on the inflow bulk (i.e. cross-section average) velocity (*U*_*b*_) and the parent artery diameter at the inlet (*D*) is in the range of *Re* = *U*_*b*_ *D*/*ν* = 238–651 with a cycle-averaged value of ∼366. The Womersley number based on the inlet vessel diameter and a waveform period of *T* ≈ 7.92 s is $Wo=D/2\sqrt{2\pi T^{-1}\nu^{-1}}\approx 3.51$. The waveform pulsatility index, $PI=\left(U_{max}-U_{min}\right)/U_{\bar{cycle}}$, is approximately 1.13. *U*_*max*_ is the peak systolic bulk velocity, *U*_*min*_ is the end diastolic bulk velocity, and $U_{\bar{cycle}}$ is the cycle-averaged bulk velocity at the inlet. The resistive index, *RI* = (*U*_*max*_ − *U*_*min*_)/*U*_*max*_ is approximately 0.63. Two steady state flow conditions, one at the systolic peak, i.e. *Re* = 651, and one at a higher Reynolds number, i.e. *Re* = 1000, are acquired using the same flow loop by bypassing the cardiac pump.

### 2.3 4D flow measurements

4D flow measurements are performed using phase contrast magnetic resonance imaging (PC-MRI). This non-intrusive measuring technique, unlike PIV and LDA, does not require optical access or deployment of tracer particles, providing all three velocity components in the volume of interest with sub-millimeter resolution. The fundamental concept of the velocity encoding is based on the relation between the fluid velocity and the MR signal phase along a magnetic field gradient. For a comprehensive review of the method and its applications, the reader is referred to [27, 29].

Flow measurements in the present study are performed at the Center for Magnetic Resonance Research (CMRR) at the University of Minnesota. A 64 channel head/neck receiver coil is used in a 3 Tesla Siemens human MRI scanner. The phase-locked measurements of the volumetric flow field are acquired for 16 phases of the cardiac cycle using the velocity acquisition sequence discussed in [29]. The full acquisition cycle is repeated twice and the velocity fields are averaged to increase the statistical accuracy. The acquisition time for each test is approximately 2.5 hours corresponding to 560 cardiac cycles. It is worth noting that *in vivo* measurements are much shorter due the fact that the period of real cardiac cycles are shorter. Details of the PC-MRI settings are given in Table 1.

**Table 1:** PC-MRI parameters.

Parameter	Value	Unit
Echo time	5.4	ms
Repetition time	492	ms
Bandwidth	465	Hz/px
Velocity encoding*	0.8	m/s
Flip angle	15	°
Bit-depth	12	−
Spatial resolution	0.6×0.6×0.6	mm^3^
Field of view	230×71×76	mm^3^
Number of scans	2	−
Signal-to-noise ratio	80	−

* along all directions.

The velocity random error, *σ*_*v*_, is estimated using the relation proposed by [30]:

$$\begin{array}{c} \sigma_{v}=\frac{\sqrt{2}}{\pi }\frac{V_{enc}}{SNR} \end{array}$$ (1)

where *V*_*enc*_ is the velocity encoding which defines the maximum measurable velocity, and *SNR* is the signal-to-noise ratio. Here, *SNR* is calculated as:

$$\begin{array}{c} SNR=\frac{<S>-<B>}{\sigma_{b}} \end{array}$$ (2)

where < *S* > is the mean of MR signal inside the wetted volume (i.e. flow region), < *B* > is the mean of the MR signal in the background (i.e. outside the flow region), and *σ*_*b*_ is the standard deviation of MR in the background. For *N* number of scans, *SNR* in the above is improved by a factor of $\sqrt{N}$ [31]. The observed random error in the velocities is approximately ∼6.8% of the cycle-averaged bulk velocity at the inlet. For alternative methods to estimate *SNR* in MRI experiments and the effect of different parameters and source of velocimetry inaccuracies see [30, 32].

### 2.4 Data pre- and post-processing

The wetted volume, i.e. lumen volume, is determined based on a selected *SNR* threshold of 8.5. The reconstructed lumen surface is dilated and eroded in order to remove any potential artifacts at the geometry’s boundaries. In order to take into account any spurious systematic spatial variation of the flow inside the region of interest due to the coil response, before and after each flow test, a flow-off state is scanned. Identical MRI acquisition parameters as used in the flow-on tests are used for this purpose. A polynomial regression is used for each velocity component to obtain the distribution of the signal when the fluid is at rest. The analytical fit to the flow-off field, which is below 1% of $U_{\bar{cycle}}$, is then subtracted from the flow-on velocity field at each phase, see [33] for further details.

After flow-off subtraction, an outlier detection method is applied to the velocity field at each phase. This filter is based on the local normalized median of velocities and the use of a varying threshold based on the local MR signal. A rejected velocity vector is then replaced with the median of the neighboring vectors. In addition, a standard median filter with a kernel size of 3×3×3, and a Gaussian filter with the same kernel size are applied to the data. It is followed by a solenoidal filter application as described in [34] to ensure that the mass conservation is globally enforced in an integral sense. The various filtering techniques applied here produced changes smaller than 10% of the unfiltered data at all points in the flow volume.

Following the velocity field filtering, the relative pressure field at each phase is reconstructed by solving the Poisson pressure equation as described in [35]. The Poisson equation is discretized and solved using isoparametric hexahedral finite elements. For the wall shear stress calculation, the unit normal vector to the treated reconstructed wall is first computed. The dot product of the vorticity vector adjacent to the wall and the local wall-normal vector is calculated to find the angle between the two. The magnitude of the vorticity vector (*ω*_*mag*_) tangent to the wall is then locally calculated. For a given dynamic viscosity, the local wall shear stress is obtained as *τ*_*w*_ = *νρω*_*mag*_|__*wall*,*tan*__.

### 2.5 Pressure measurements

**Fig 3:**
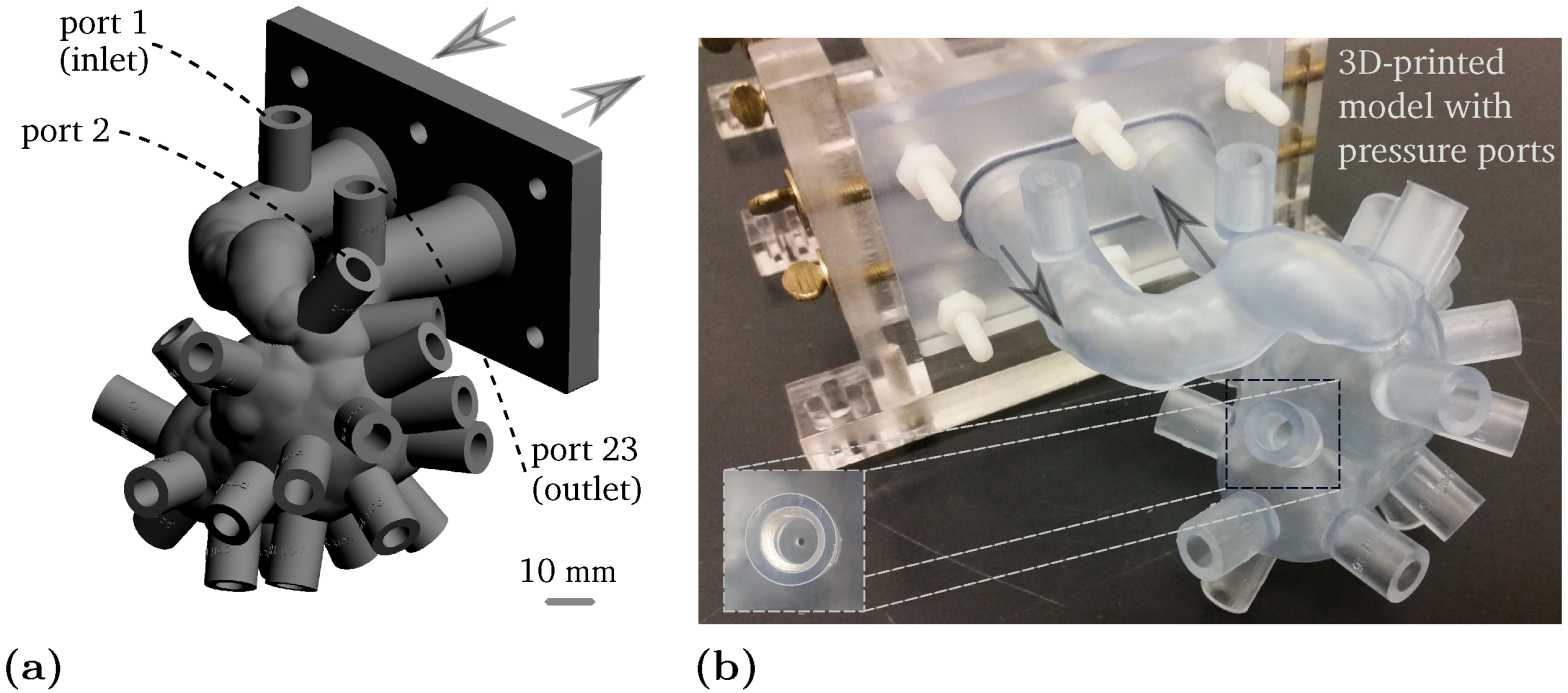
(a) Aneurysm model with 23 ports for the pressure measurements. (b) 3D-printed phantom. The pressure reading holes have an inner diameter of ∼1 mm. The arrows represent the flow direction.

For the purpose of pressure measurements, a phantom with 23 ports is designed and built in the same way as the previous phantom. Holes for the pressure ports are drilled into the phantom with an inner diameter of approximately 1 mm. The model geometry and the fabricated phantom are shown in Fig 3(a) and 3(b), respectively. A Validyne diaphragm transducer (DP-15) is used for the pressure waveform measurement at the aneurysm inlet and for the pressure drop measurements over the aneurysm sac. A Validyne sine wave carrier demodulator (CD-15) is used to acquire the high fidelity analog signal.

To achieve the highest dynamic range, a diaphragm with a high sensitivity range of 0-0.86 kPa is used to capture the differential pressure, i.e. between the port at the inlet and other ports. A diaphragm in the range of 0-8.6 kPa is used to measure the pressure difference between the inlet port and ambient. According to the manufacturer, pressure is measured with an accuracy of ±0.25% of the full scale range with a dynamic response of 1 kHz. The pressure transducer has been calibrated against a Rosemount 3051S pressure transmitter over the range of expected experimental pressures. The phantom is connected to the same flow loop using thick tubing with minimal compliance. The pressure and flow signals are recorded for approximately 55 cardiac cycles at a sampling rate of 200 Hz.

## 3 Results

### 3.1 Flow waveform evaluation

**Fig 4:**
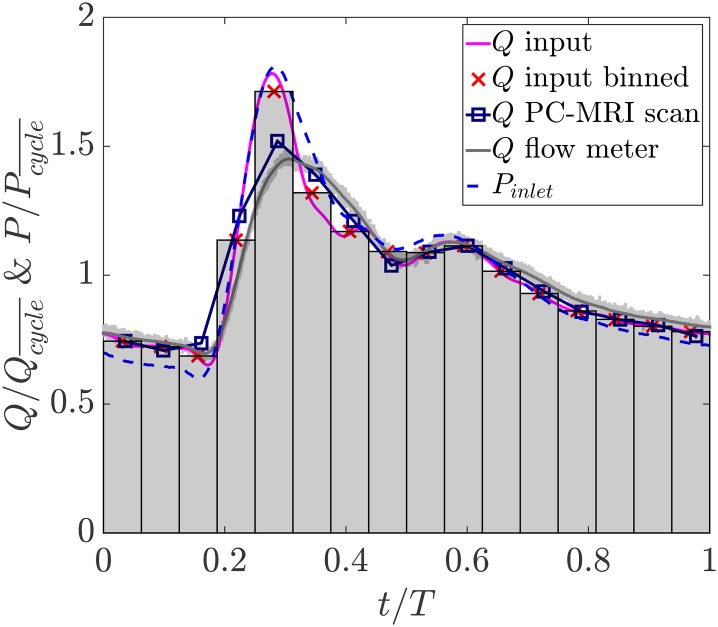
Input flow rate waveform along with the binned input waveform, and the inflow waveform reconstructed from PC-MRI at the inlet of the aneurysm. The flow rate and pressure waveforms measured upstream of the aneurysm using the ultrasonic flow meter and pressure transducer respectively are shown for comparison.

The inflow waveforms are shown in Fig 4. The bins represent 16 acquisitions over the cardiac cycle with the cross symbols showing the flow rate averaged within each bin. The flow rate waveform reconstructed from PC-MRI at the inflow vessel is in good agreement with the imposed input in which all features including the dicrotic notch are resolved. Quantitatively, there is a maximum mismatch of approximately 10% at the systolic peak. This is likely since due to difficulties in resolving the systolic velocity gradient at the wall, a fraction of the velocity field near the wall is not well captured. The flow rate measured by the ultrasonic flow meter is also provided. The light gray lines each represent one cardiac cycle measured by the flow meter over the course of the experiment. A minimal cycle to cycle variation is observed in the generated flow waveform. The standard deviation band of variations has a maximum value of 2% with respect to the ensemble average flow rate showing a repeatable waveform generated by the cardiac pump. Finally, the inlet pressure waveform measured as described in Section 2.5 is also shown. $P_{\bar{cycle}}$ is the cycle-averaged pressure at the inlet and $Q_{\bar{cycle}}$ is the cycle-averaged flow rate.

### 3.2 Flow structure and statistics

**Fig 5:**
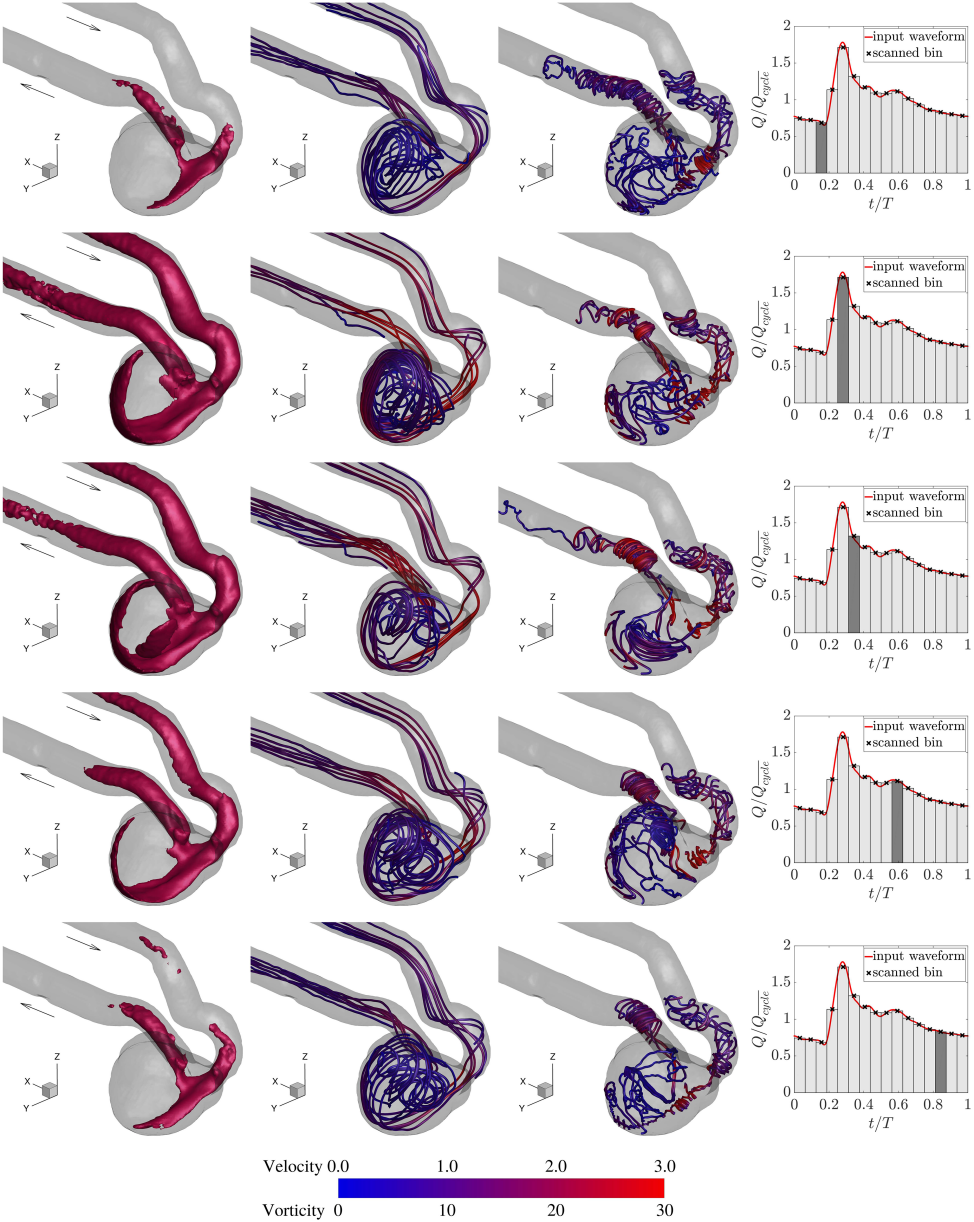
Columns from left to right: Iso-surface of the velocity magnitude at $1.5U_{\bar{cycle}}$, streamlines, vortex lines, and the measurement phase. Five measurement phases from top to bottom rows are early systole, systolic peak, late systole, dicrotic notch, and late diastole, respectively. Streamlines are colored by the normalized velocity magnitude ($U_{mag}/U_{\bar{cycle}}$), and vortex lines are colored by the normalized vorticity magnitude ($\omega_{mag}D/U_{\bar{cycle}}$).

To show the evolution of the flow pattern in the aneurysm sac, the iso-surface of velocity magnitude at a threshold level of $1.5U_{\bar{cycle}}$ is depicted in Fig 5 along with the corresponding streamlines and vortex lines for five cardiac phases. The iso-surface of the velocity shows the region where the flow travels faster than the bulk velocity in the vessel. Due to the stenosis at the parent artery right before the aneurysm neck, the flow accelerates while entering the aneurysm sac in such a way that the jetting flow reaches the aneurysm apex, impinges on the distal wall, and rolls back. This happens consistently for approximately half of the cardiac cycle.

Highly three-dimensional vortical flow structures inside the aneurysm sac are evident from both the streamlines and vortex lines. Streamlines that are tangent to the local velocity vectors are useful to examine the complex flow field. The vortex lines that everywhere are tangent to the vorticity field are essentially the continuity of vortex filaments. Due to the anatomy of the inflow vessel and the location of the aneurysm with respect to the parent artery, the incoming flow to the aneurysm sac is somewhat vortical and helicoidal. The vortical flow entering the aneurysm sac induces a single large vortex and other small vortices that can be identified from the streamlines. The vortex lines in the sac imply the existence of a few vortices of different rotational strength that can be identified with the highest strength near the aneurysm neck and along the wall proximal to the neck. Vortex lines tend to align with the vessel at the aneurysm inflow and outflow. Although the shape and location of vortices may vary over the cardiac cycle, the general flow structure is preserved.

**Fig 6:**
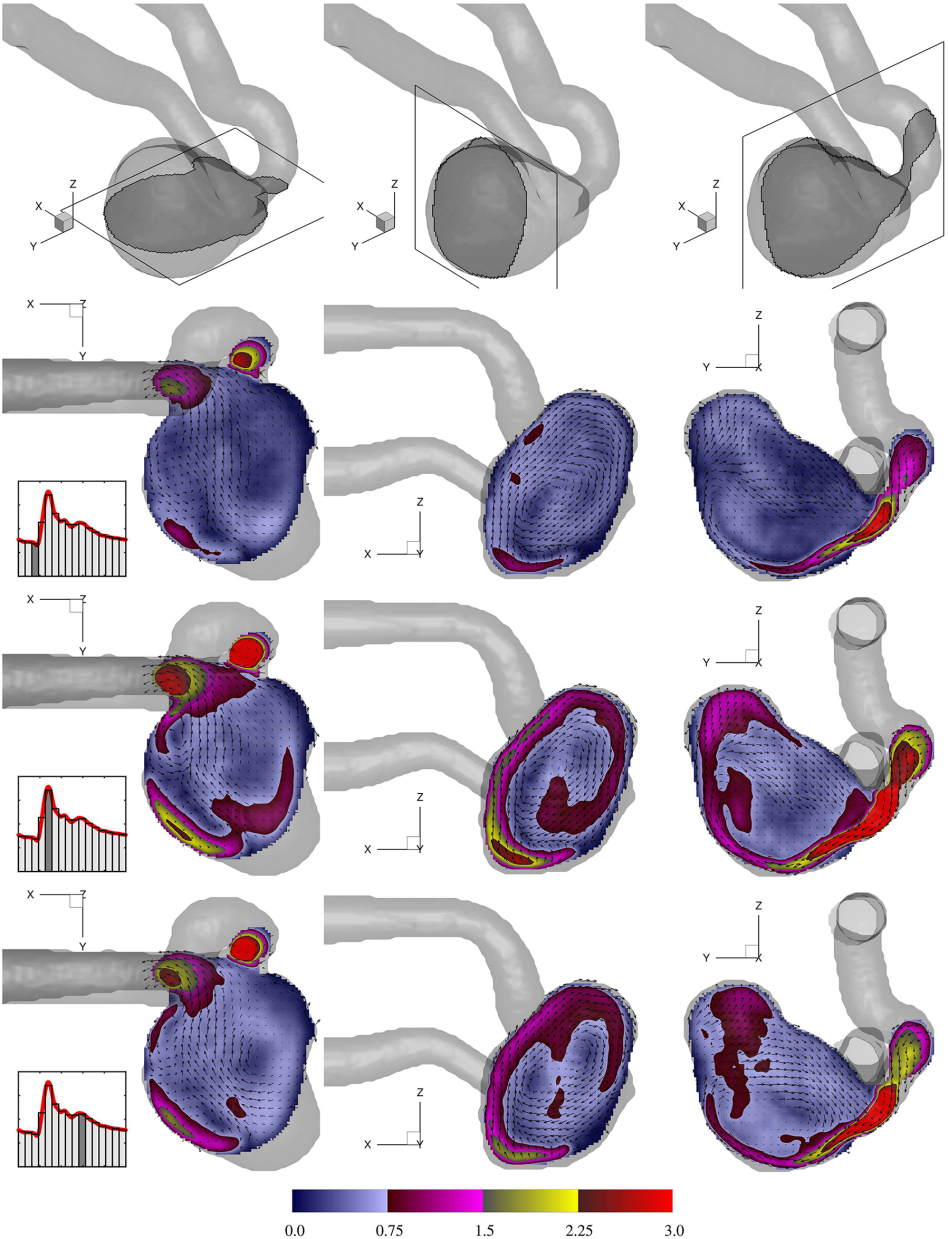
Normalized velocity magnitude ($U_{mag}/U_{\bar{cycle}}$) at three selected cut planes in the aneurysm sac. Each row shows one cardiac phase. Note that for better visibility, every third vector in each direction is shown.

To have a clearer view of the flow pattern, velocity magnitude in orthogonal slices with the corresponding vector fields are shown in Fig 6. The highly rotational nature of the flow is evident in all directions. The cross planes of the velocity iso-surface shown in Fig 5 can be seen here showing the penetration of the fast moving fluid into the sac. This constitutes the dominant flow structure in all three directions. The main vortex is induced by the fast inflow jetting into the sac while other vortices are formed due to the shear layer between the high speed jet and low speed flow residing in the aneurysm sac. Such coexisting vortices are more easily identified through the *X* − *Y* cross sections.

**Fig 7:**
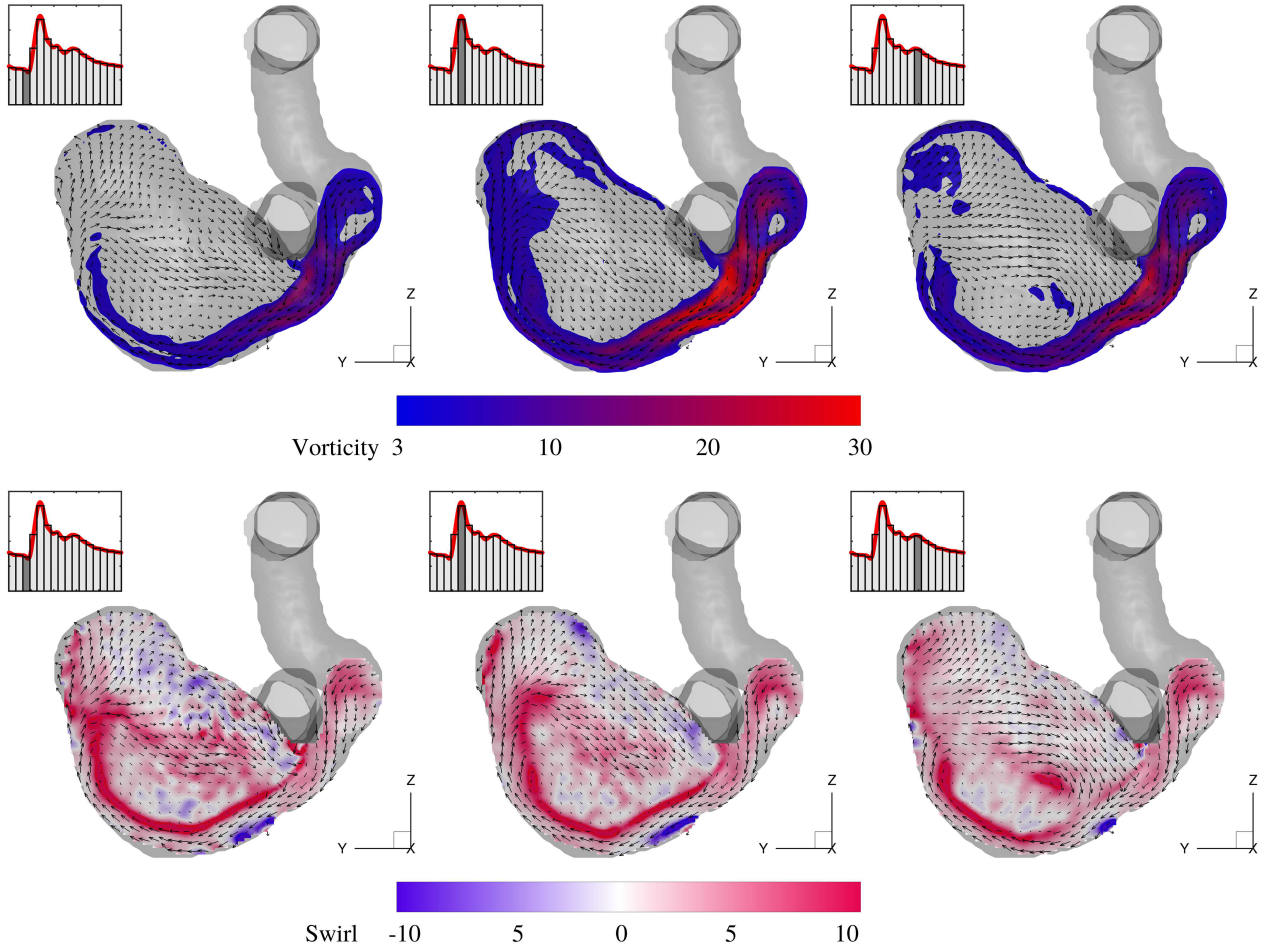
Normalized vorticity magnitude ($\omega_{mag}D/U_{\bar{cycle}}$), and normalized swirl (*SD*) for three cardiac phases at the same *Y* − *Z* slice shown in Fig 6. Every third vector in each direction is shown.

The evolution of vorticity magnitude and the corresponding swirl is shown in a *Y* − *Z* cut plane in Fig 7 for three cardiac phases. For a better visualization, a threshold level of 3.0 for the normalized vorticity magnitude is used to blank the low vortical regions. The high vorticity level at the shear layer extends towards the distal wall and progressively reaches the tip of the aneurysm dome and beyond. Swirl (also termed helicity) is calculated as $S=\vec{U}\cdot \vec{\omega }/{\left|\vec{U}\right|}^{2}$ is a scalar quantity that shows the alignment of the vorticity vector with the local velocity vector. Swirling motions persist in a large volume of the sac with the highest strength at the interface of the fast incoming jet and the low speed recirculation region. Swirl maps are useful as they show the regions (e.g. the middle of the sac) where the vorticity level is low but is parallel to the local velocity, whereas the vorticity maps mostly pick the shearing regions. Due to the anatomy of the parent artery, the incoming jet flow follows the aneurysm wall and the shear layer remains attached to it.

**Fig 8:**
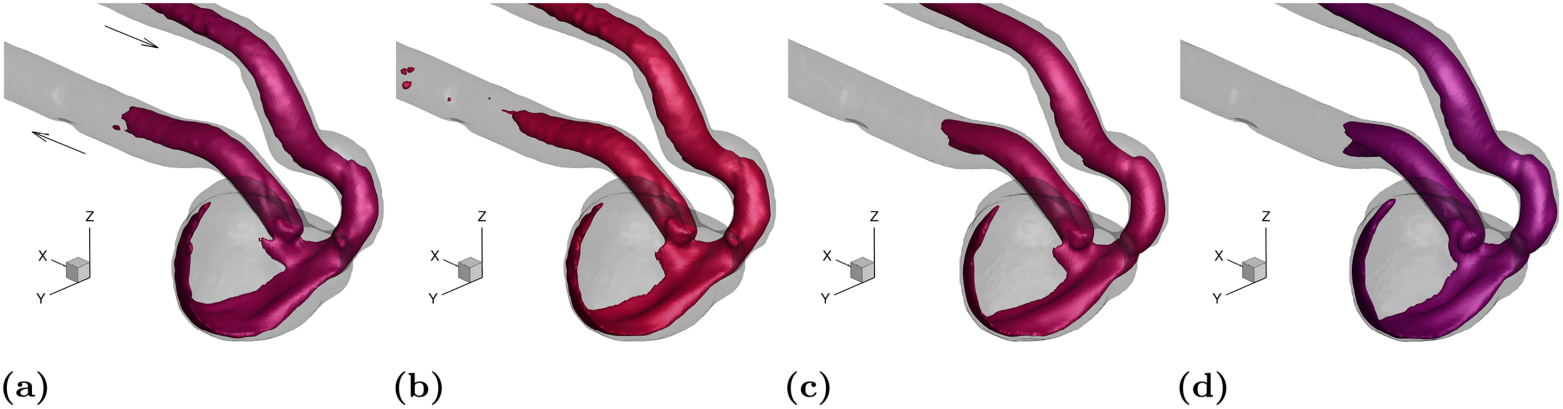
Flow structure at: (a) systole (0.25 ≲ *t*/*T* ≲ 0.31); (b) cycle-averaged state (0 ≤ *t*/*T* ≤ 1); (c) steady state with the Reynolds number at the systolic peak, *Re* = 651; (d) steady state at *Re* = 1000. The velocity iso-surface thresholds are $1.9U_{\bar{cycle}}$, $1.1U_{\bar{cycle}}$, $1.9U_{\bar{cycle}}$, and $2.9U_{\bar{cycle}}$, respectively.

To show the persistence of the flow pattern in the aneurysm sac, iso-surfaces of the velocity magnitude at the systolic peak and cycle-averaged states are shown in Fig 8(a) and 8(b), respectively. The thresholds for velocity iso-surfaces are chosen proportional to the inflow flow rate. The flow structure under systolic and cycle-averaged conditions looks very similar suggesting that the main flow feature resides in the sac for a number of phases and hence in an average sense it outweighs the low speed features in the rest of the cycle. In addition, two steady state flow measurements at the systolic peak, i.e. *Re* = 651, and at a higher Reynolds number, i.e. *Re* = 1000 are shown in Fig 8(c) and 8(d), respectively. As can be seen, the flow pattern at both steady conditions closely resembles the systolic and cycle-averaged cases. This suggests that the same flow structure resides in the sac regardless of the measurement phase and Reynolds number. It seems likely then the flow is mostly dictated by the aneurysm geometry (sac size, aspect ratio, and location with respect to the parent artery) with only weak sensitivity to the pulsatility of the incoming waveform. This is valid at least for the inflow conditions examined here.

**Fig 9:**
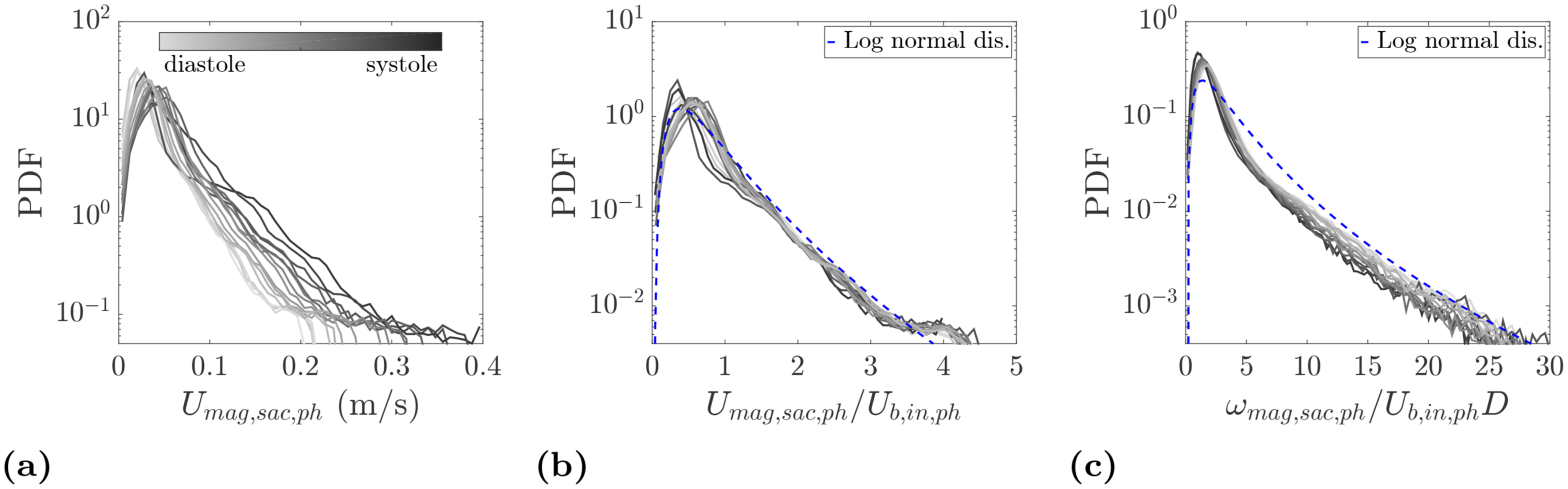
(a) PDFs of the velocity magnitude in the aneurysm sac. (b) PDFs of the normalized velocity magnitude using the phase-locked bulk velocity at the inlet, *U*_*b*,*in*,*ph*_. For log-normal distribution, *σ* = *σ*_*U*_*mag*,*sac*,*cycle*__ and *μ* = < *U*_*mag*,*sac*,*cycle*_ > are calculated using all velocity points inside the sac over all phases. (c) PDFs of the normalized vorticity magnitude in the aneurysm sac. PDFs are color coded with the measurement phase from diastole (light gray) to systole (dark gray).

To have a more quantitative comparison of velocities at different phases, probability density functions (PDFs) of the velocity magnitude are shown in physical and normalized units in Fig 9(a) and 9(b), respectively. Naturally, a wider velocity range is expected at the systole and its neighboring phases. However, the distributions collapse when velocities at each phase are normalized by the inlet bulk velocity at that phase, *U*_*b*,*in*,*ph*_. This provides further evidence of the existence of a persistent flow pattern in the aneurysm sac shown in Fig 8. Another point to note in Fig 9(b) is the presence of a log-normal behavior. In this figure, the standard deviation (*σ*) and ensemble average (*μ*) of the velocities inside the aneurysm sac over the entire cycle are used to plot a log-normal distribution. At each phase more than 75% of the velocity vectors have a magnitude between zero and the inlet bulk velocity at that phase. The rest of the velocity probability corresponds to the fast traveling fluid due to the incoming jet into the aneurysm sac. The most probable events at each phase occur at approximately 0.5*U*_*b*,*in*,*ph*_, which is consistent with the mode of a log-normal distribution, *μ*(1 + (*σ*/*μ*)^2^)^−3/2^ [36]. The use of the same velocity scale also leads to a good collapse of PDFs of the vorticity magnitude in the sac as shown in Fig 9(c). The same applies to the PDFs of individual velocity and vorticity components.

### 3.3 Wall shear stress distribution

**Fig 10:**
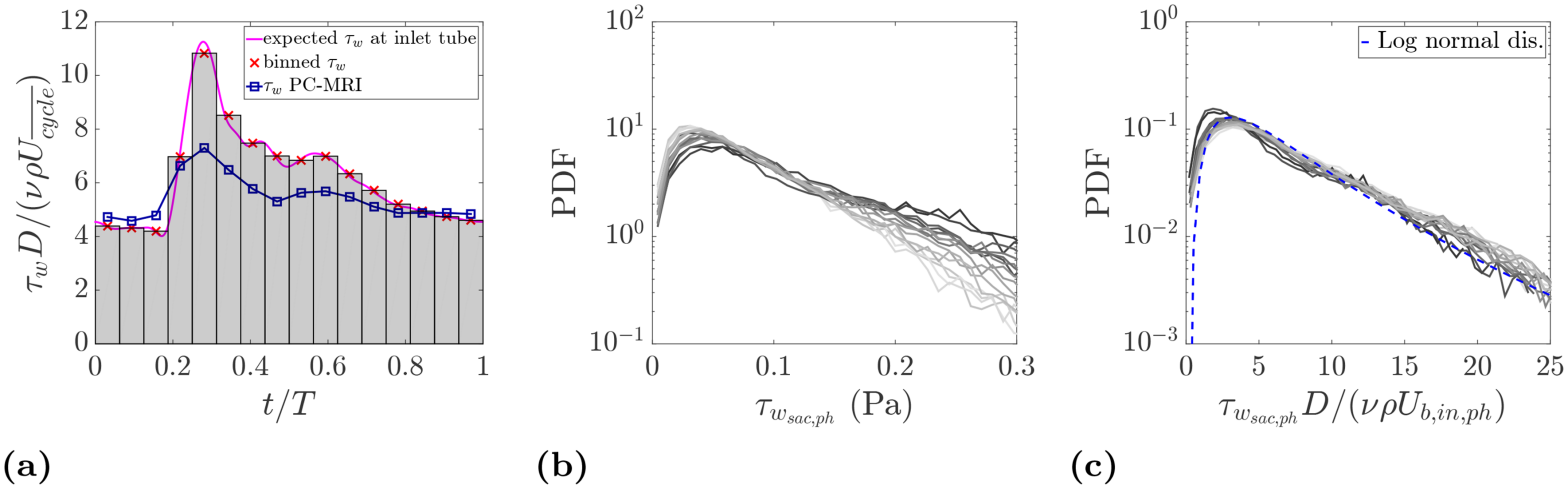
(a) Estimation of the wall shear stress at the tube upstream the inflow vessel. The expected wall shear stress is calculated from the Womersley solution. (b) PDFs of the wall shear stress at the aneurysm sac. (c) PDFs of the normalized wall shear stress using the inlet bulk velocity. The color coding in PDFs is the same as in Fig 9.

**Fig 11:**
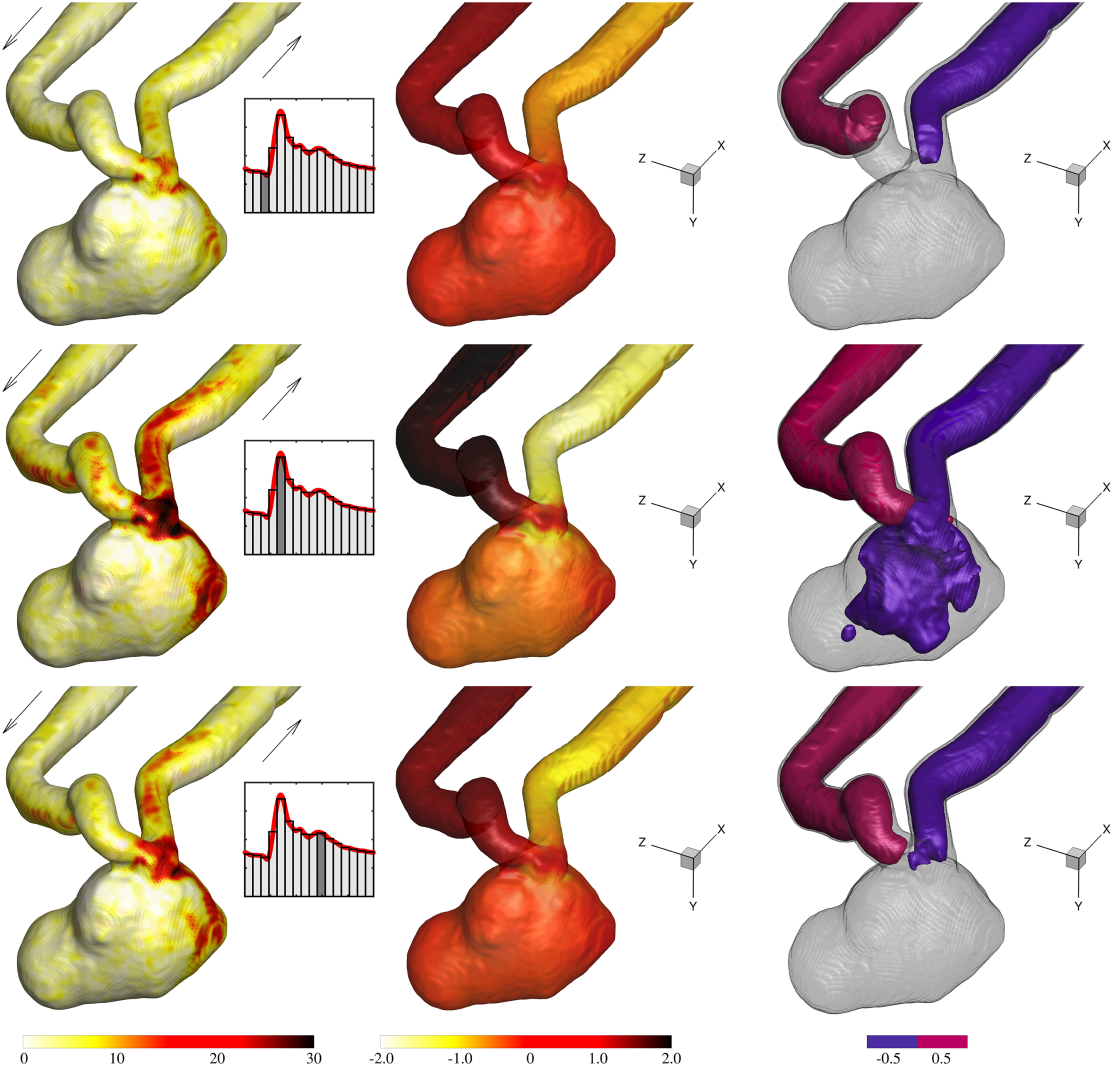
Columns from left to right: Normalized shear stress at the wall, $\tau_{w}D/\left(\nu \rho U_{\bar{cycle}}\right)$, normalized relative pressure at the wall, $P_{w}/P_{\bar{cycle}}$, and iso-surface of the relative pressure at threshold levels of $\pm 0.5P/P_{\bar{cycle}}$.

The spatial resolution of the experimental technique compares well with previous *in vitro* and *in vivo* studies, however, it may not be sufficient to accurately capture the gradients at the wall where the strongest change in the velocity usually occur. First, we checked the accuracy of the wall shear stress (WSS) calculation in the straight inlet tube under well defined geometry and flow condition, using an harmonic flow waveform decomposition [25]. For this purpose, the 22 sinusoidal waves (harmonics) composing the inflow waveform given by [15] are used. Fig 10(a) shows the overall WSS obtained by the superimposition of the stresses calculated at each harmonic. The estimated wall shear stress, depending on the cardiac phase, is up to 30% different from the expected true value. The mismatch is most pronounced at the systolic peak. Similar to the flow rate, this is again due to the inability to fully resolve the velocity gradient close to the wall. Therefore, the calculated shear stress is to be considered an approximation of the actual WSS. Nevertheless, this approximation is insightful as it images the WSS distribution along the parent artery and over the aneurysm sac showing the magnitude of stresses relative to each other. Fig 11 shows the WSS map on the aneurysm for three cardiac phases that indicate regions with elevated shear. The highest shear is found at the aneurysm neck where the flow is accelerated due to the stenosis in the parent artery. In addition, the aneurysm sac experiences an increased level of shear where the incoming jet impinges. PDFs of the shear stress estimated at the aneurysm wall are shown in Fig 10(b) and 10(c) in physical and normalized units, respectively. A similar trend to the velocity and vorticity PDFs is observed here. The use of the inlet bulk velocity leads to a good collapse of the PDFs.

### 3.4 Pressure distribution

Fig 11 shows the iso-surface of the normalized relative pressure for three cardiac phases where it decreases from the inflow to the outflow vessel. At the systolic peak, a relatively large low pressure region affects the aneurysm sac due to a stronger jet being injected into the sac. Hence, a stronger pressure gradient across the induced vortex is expected. The low pressure region is located at/near the core of the large vortex. The pressure at the wall is also shown in Fig 11. Pressure in the parent artery is reduced towards the aneurysm neck but is slightly elevated at the wall proximal to the neck and at the dome where the jet impingement is located. These regions are easier to be recognized in the systole where there is a higher pressure difference in the sac.

**Fig 12:**
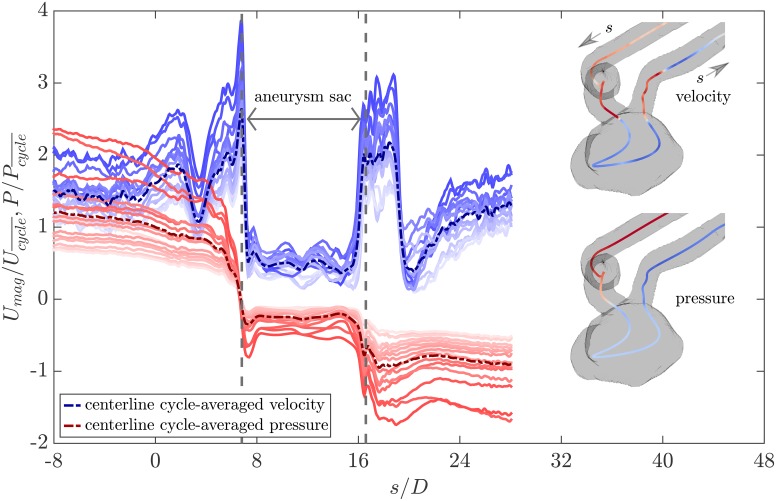
Normalized relative pressure, $P/P_{\bar{cycle}}$, and normalized velocity magnitude, $U_{mag}/U_{\bar{cycle}}$, at the phantom centerline. Each blue line represents velocity distribution at the centerline in one phase from diastole (light blue) to systole (dark blue) with the dashed blue line showing the cycle-averaged velocity. The red color coding shows the pressure distribution at the centerline in the same fashion. The centerlines shown at the top right and bottom right are respectively colored by the local systolic velocity and systolic pressure from blue (low) to red (high).

Fig 12 shows the normalized relative pressure along with the normalized velocity magnitude at the centerline of the phantom for different cardiac phases as well as the cycle average. The centerline is segmented from the wetted volume using SimVascular [37]. The origin of the centerline coordinate is at the aneurysm inlet and the curvilinear abscissa (*s*) is normalized by the vessel diameter at the inlet (See Fig 1(a)). As expected, velocity remains almost constant with a slight increase towards the aneurysm inlet (-8 ≲ *s*/*D* ≲ 0) where the pressure monotonically decreases. The sharp decrease and increase in the velocity in the range of 1 ≲ *s*/*D* ≲ 5 corresponds to the sharp bend of the parent artery. Inspection of the velocity magnitude at the corresponding cross planes shows skewed velocity maps which explains the oscillations of the centerline velocity in this region. The pressure is more uniformly distributed at these planes and therefore no sharp pressure variation is observed. When the flow approaches the stenosis location at the aneurysm neck (*s*/*D* ≈ 6.8), the velocity spikes and the pressure drops sharply. There is a mild pressure recovery downstream the stenosis (6.8 ≲ *s*/*D* ≲ 8) where the expansion to the aneurysm sac happens. In the aneurysm sac (6.8 ≲ *s*/*D* ≲ 16.6), the pressure and velocity remain almost constant along the centerline as it follows the recirculation region. Near the aneurysm outlet (15.8 ≲ *s*/*D* ≲ 16.6), due to the contraction, velocity increases quickly and pressure decreases accordingly. At the outlet of the parent artery, the flow is helical and vortical and hence the velocity map is skewed and the centerline sees oscillations. Eventually, through the downstream vessel (*s*/*D* ≳ 23), velocity increases monotonically and pressure decreases mildly along the straightened outflow.

**Fig 13:**
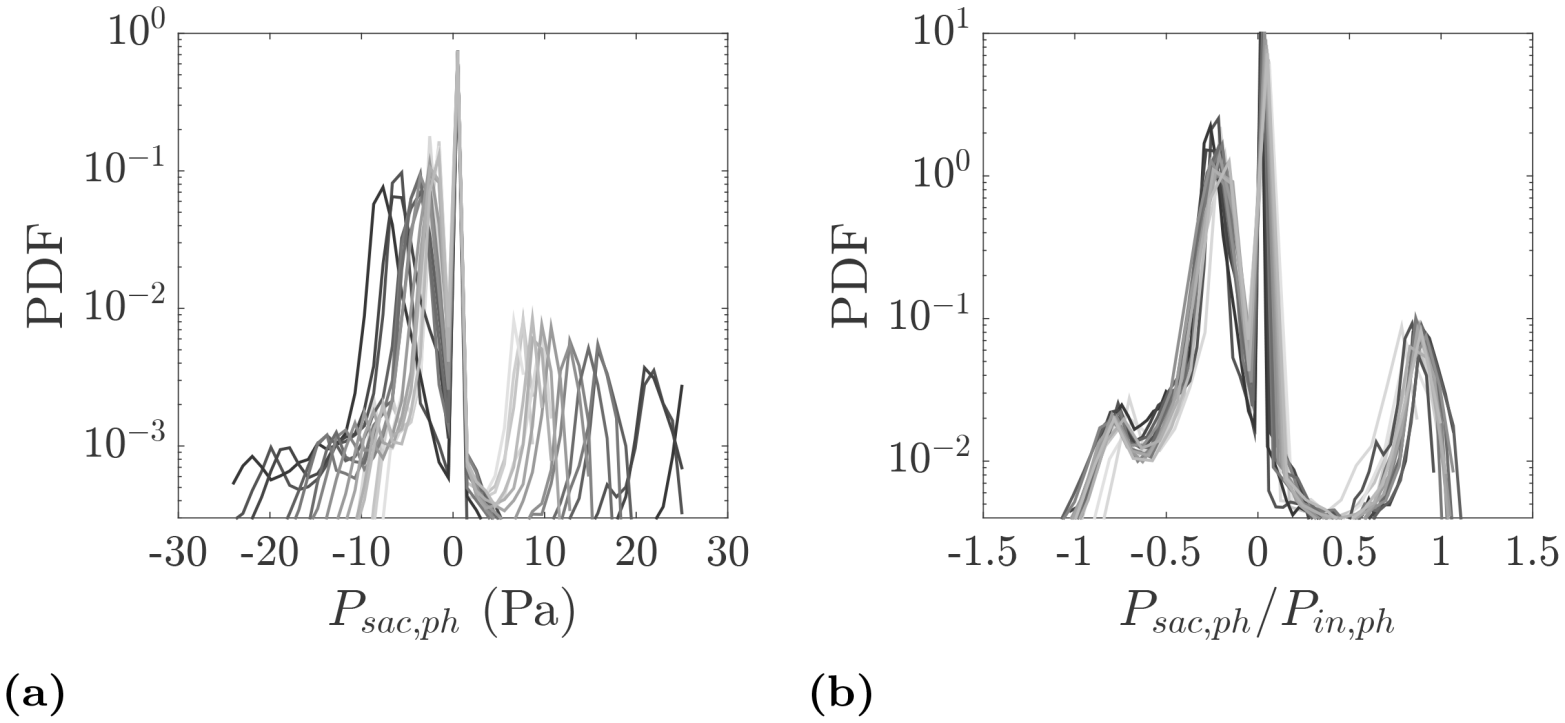
(a) PDFs of the relative pressure inside the aneurysm sac. (b) PDFs of the normalized relative pressure using phase-locked pressure at the inlet. The color coding is the same as in Fig 9.

In order to further quantify the pressure distribution in the sac, PDFs of the pressure are shown in Fig 13 in physical and normalized units. In Fig 13(a), for phases with smaller inlet bulk velocity, a smaller range of pressure is noticed whereas close to systole a wider range of values is observed. The inspection of the velocity and pressure fields indicates that the peak of positive/zero pressure is associated with two main regions: the impingement of the jet into the sac wall and the stagnation region at the aneurysm dome. The wider and larger negative pressure peak in the PDFs relates to the remainder of the aneurysm volume. The use of the inlet pressure scaling collapses the PDFs as shown in Fig 13(b) which again indicates that the essential hemodynamics features are maintained during the cardiac cycle.

**Fig 14:**
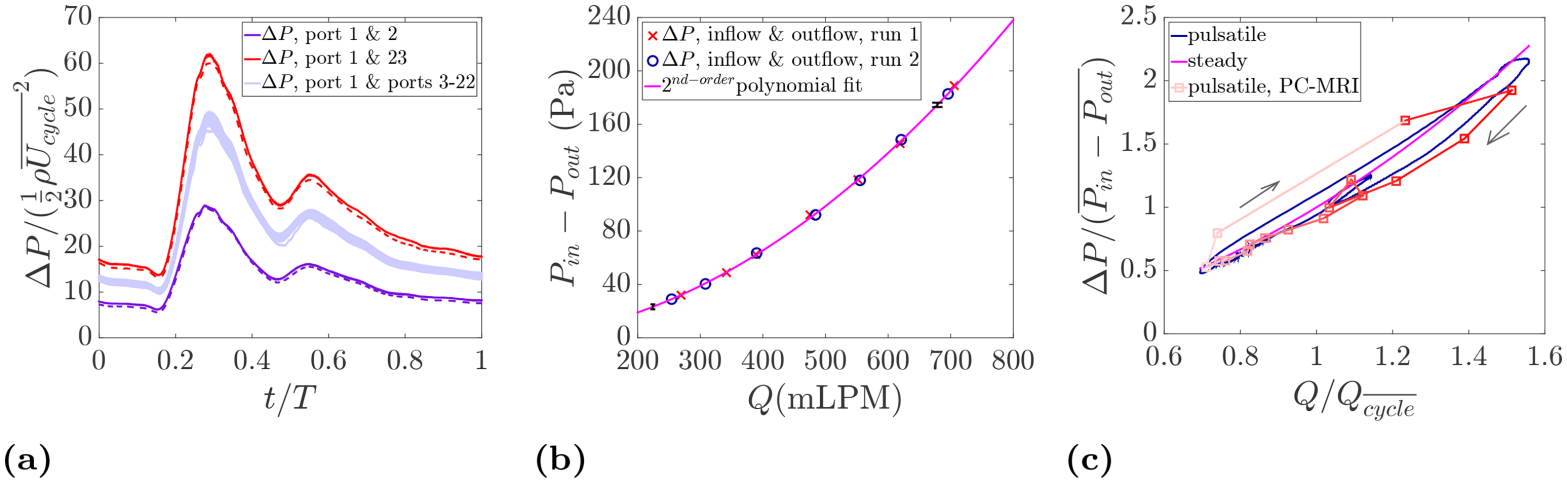
(a) Pressure drop along the inflow vessel (between port 1 & 2), over the aneurysm sac (between port 1 and ports 3-22), between the inflow and outflow (between port 1 & 23). The dashed lines indicate the second run of the measurement. Each waveform is an ensemble average of 55 cardiac cycles. (b) Pressure drop between the inlet (port 1) and the outlet (port 23) in the steady state flow condition. (c) Evolution of the pressure drop between the inlet and the outlet in the cardiac cycle measured by the pressure transducer and PC-MRI.

To further investigate the pressure drop evolution, pressure measurements detailed in Section 2.5 are shown in Fig 14. Pressure loss over an aneurysm sac could be an important factor as it is an indication of energy dissipation and could be used as a metric for aneurysm growth and rupture risk [38, 39]. The temporal variation of the normalized pressure drop is given in Fig 14(a). The pressure drop over the inflow parent artery (between port 1 and port 2, see Fig 3(a)) is the smallest since port 2 is located just before the aneurysm entrance. The pressure difference between the inlet (port 1) and the ports on the aneurysm sac (from 3 to 22) experiences a larger pressure drop as these ports are located past the stenosis and the flow is expanded. Within the measurement uncertainty, the wall pressure on the aneurysm sac experiences a very similar pattern with very small variation from one port to another. The small variation on the wall pressure map has also been noted earlier in Fig 11. Finally, the highest pressure drop as expected is observed between the inlet and the outlet (port 1 and 23) where the pressure over the entire sac and the parent vessel is measured. In addition, the pressure difference between port 1 and 23 is measured in the steady state condition for the range of flow rates expected in the cardiac cycle. A second order polynomial regression is obtained as shown in Fig 14(b) yielding the expected quadratic relation between pressure drop and flow rate.

In Fig 14(c), the pressure drop measured by the pressure traducer in the pulsatile and steady states are compared with the one computed from the PC-MRI. As can be seen, a very good agreement is noticed especially between the pulsatile cases. The steady state plot also predicts the trend in an average sense. In order to better understand the pressure behavior in terms of the input flow rate, a color coding similar to Fig 12 is used along with arrows indicating the direction of time. End of diastole is located at the bottom left of the plot where the pressure and flow rate are at the minimum level. The pressure drop increases along with the flow rate reaching the systolic peak, and decreases towards diastole. The interesting point to notice in the pressure-flow diagram is the formation of a small loop inside the large loop that corresponds to an increase in the pressure drop due to the dicrotic notch. Although only 16 phases are measured by PC-MRI, all flow features including the inner loop are well captured.

### 3.5 A brief comparison with 2012 CFD Challenge

**Fig 15:**
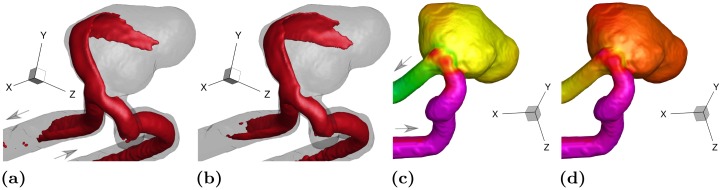
(a) Cycle-averaged velocity pattern at a threshold level of $1.1U_{\bar{cycle}}$ that is equivalavnt to 0.3 m/s used in [15]. (b) Systolic peak velocity pattern at a threshold level of $1.9U_{\bar{cycle}}$ that corresponds to 0.5 m/s used in the computational studies. (c) Cycle-averaged wall pressure map. The color bar range used here is ∼2.0 cycle-averaged pressure drop between the inlet and outlet of the aneurysm that corresponds to 75-90 mmHg used in the computations in [15]. (d) Wall pressure map at the systolic peak. The color bar range is ∼5.3 cycle-averaged pressure drop between the inlet and outlet of the aneurysm corresponding to 80-120 mmHg used in the computational studies.

To determine exactly how our experimental results compare with the computational simulations reported in the 2012 CFD Challenge [15], a comprehensive comparative study would be needed which is beyond the scope of the present work. Here we present a brief comparison in terms of the cycle-averaged and systolic velocity magnitude and wall pressure, as reported in [15]. Velocity magnitude iso-surfaces for the cycle-averaged and systolic peak states are respectively shown in Fig 15(a) and 15(b) for the same threshold levels and angle of view as used in [15]. For comparison, readers are referred to Figs 10 and 11 in [15] with a focus on the simulations labeled as “W” and “X” that were carried out with particularly high spatial and temporal resolution and thus considered closest to a “gold standard” by [15]. The cycle-averaged measurements are in close agreement with most of the simulations. On the other hand, the same iso-surface at the systolic peak shows significant variability among the CFD solutions in terms of penetration of the jetting flow into the aneurysm sac. In addition, flow instabilities are noticed in some cases (visible in the wobbling shape of the iso-surfaces) but not in others. According to [15] the disagreement among different solutions could be due to insufficient temporal resolution in the calculations. We remark that our data is the result of a phase-average process, and therefore instantaneous flow instabilities (if present) would not be captured.

Fig 15(c) and 15(d) display the cycle-averaged and systolic pressure distribution at the wall, respectively. For comparison, readers are referred to Figs 6 and 7 in [15] with a particular focus on “W” and “X” simulations. The experimental measurements capture the fine details reproduced by the CFD simulations, and there is good quantitative agreement with most of the numerical solutions in terms of cycle-averaged pressure. On the other hand, the systolic distributions show wider disparity, with most of the simulations indicating higher pressure drop compared to the measurements here. Considering the median of the 27 solutions in [15], the inlet-to-outlet pressure drop at systole was found to be approximately 2.7 times the cycle-averaged pressure drop. Our MRI-based pressure measurements indicate that the ratio between the systolic and cycle-averaged pressure drop is ∼2.0 which is in agreement with the benchmark measurements using the pressure transducer which gives ∼2.2.

The discrepancies at the systolic peak confirm that the unsteadiness during the flow acceleration phase is challenging to reproduce numerically [15]. It is also worth underscoring the role of the stenosis in the parent artery right before the aneurysm sac. There, the cross-section area of the vessel is reduced by ∼70%, with a consequently strong and localized pressure drop. Therefore, even small differences between the fabricated model and the geometry used in the simulations may affect the comparison. The high resolution stereo-lithography and the scaling factor of 2.0 in the present study were aimed to mitigate this effect, but manufacturing imperfections cannot be completely ruled out. However, if these had a strong impact at systole, one would expect them to also affect the cycle-averaged results, for which instead the agreement with CFD is satisfactory. Finally, we remark that in the 2012 CFD Challenge, aside from the prescribed pulsatile flow rates, there were no other constraints imposed on the inflow and outflow boundary conditions. It is therefore possible that different boundary conditions with respect to the present measurements account for some of the disagreement.

## 4 Discussions and concluding remarks

4D flow measurements in a giant intracranial aneurysm are performed using PC-MRI under pulsatile and steady inflow conditions. In addition, the relative pressure is reconstructed from velocities through a Poisson pressure equation. The evolution of the flow structure and the probability density functions of the velocity magnitude, vorticity magnitude, relative pressure, and wall shear stress show a flow configuration that is largely preserved during the cardiac cycle and is not significantly affected by the pulsatility. These findings suggest that the flow pattern is mostly dictated by the aneurysm sac size and geometry and is quite independent of the input conditions. Normalized pressure drop over the aneurysm sac measured at different locations of the sac using a pressure transducer is also found in good agreement with the calculated pressure distribution. The wall pressure and wall shear stress are elevated near the aneurysm neck and near the aneurysm wall where the incoming jet impinges.

A major goal of the study was to demonstrate the ability of *in vitro* 4D flow MRI to fully resolve the spatio-temporal flow features needed to characterize the hemodynamics. Previously, MRI velocimetry has been used *in vivo* to characterize blood flow in intracranial aneurysms (e.g. [40]), but limitations in resolution, SNR, and possible artifacts associated to patient scanning prevent the unambiguous determination of all flow parameters. There, the comparison with CFD simulations does not completely alleviate this point, due to inherent uncertainties in the numerical modeling as shown in [15]. *In vitro* MRI applications have been reported (for example [20]), but the resolution of the detailed flow features is hindered by the relatively small dimension of the aneurysm itself. Isoda et al. (2006) [41] scaled up the phantom but only performed a qualitative analysis of the flow topology. The present study demonstrates how state-of-the-art 4D flow MRI on scaled-up *in vitro* models can provide a full description of the fluid flow, both for the velocity field and derived quantities (such as vorticity, wall shear stress, and pressure field). By scaling up the geometry and matching physiological Reynolds and Womersley numbers, we achieve a resolution of about one hundred vectors across the aneurysm sac, while maintaining a high SNR and therefore high precision.

Such high resolution and accuracy of the measurements is necessary both for understanding the hemodynamics and for providing a reliable source of validation to CFD solutions. It is in this perspective that we chose an aneurysm that was previously studied by numerous groups, but with the only validation of the inlet-outlet pressure drop. In particular, we carried out a comparison with the results of the 2012 CFD Challenge simulations [15]. In general, a good agreement is observed between experiments and computations for the cycle-averaged solution. On the other hand, the discrepancies at the systolic peak are, in many cases, significant both in terms of high velocity structures and wall pressure distribution. This might indicate an inherent difficulty in accurately simulating the unsteady/unstable flow during the acceleration phase of the cardiac cycle in this complex geometry. More detailed topological flow features in the simulations, as well as possible effects of inflow/outflow conditions, could not be quantified here and should be addressed in future studies.

The quasi-steady nature of the flow found in our measurements could have significant hemodynamic implications. The mechanical stimuli (in terms of pressure and shear stress) imparted to the wall by the blood flow persist throughout the cardiac cycle, with the oscillatory inflow mildly varying them in magnitude. This is an inherently different situation with respect to healthy arterial blood flow, where the temporal variation of the flow features (for the same Reynolds and Womersley numbers) would be stronger. Determining the role of this quasi-steadiness in the aneurysm formation and progression is beyond the scope of the present work, and would require evidence from multiple cases. Nevertheless, the clear indication is that here the vessel geometry dominates the hemodynamics, as compared to the specific inflow waveform.

As mentioned in Section 1, a cavity flow mode exhibiting stable features during most of the cardiac cycle has been associated with side-wall aneurysms (i.e. originating from the side of the artery as in the present case, rather than at a branch point) when the aneurysm number is *An* < 1. It is reminded that the Aneurysm number is defined as *An* = *PIW*_*a*_/*D*_*a*_ where *PI* is the pulsatility index defined earlier and *W*_*a*_ and *D*_*a*_ are the aneurysm neck width and parent artery diameter, respectively [12, 14, 42]. In the present case, we have *An* ≈ 0.63, and indeed we retrieve a quasi-steady behavior with flow features persisting throughout the cycle. This is confirmed, in our study, through the main flow statistics (velocity, vorticity, pressure, and wall shear stress) that collapse remarkably well for all phases in the cardiac cycle when normalized by the relevant quantity based on the inflow at each phase. The quasi-steady nature of the flow is confirmed by the fact that quantitatively similar features are found for the steady inflow case. Although previous experimental studies have shown stable vortical patterns in intracranial aneurysms, e.g. [18, 43], to the best of our knowledge this is the first time that a quantitative account of such behavior is given based on the full volumetric flow information. We also note that the aneurysm number has been proposed as a criterion to predict the lesion evolution, with *An* > 1 being an indication of higher rupture risk [12]. In this case we have *An* < 1, and yet the aneurysm was documented to have grown significantly and ruptured shortly after treatment [24]. However, as also pointed out by [12], the aneurysm number does not include all the important geometrical parameters. Indeed, the sheer size of the present giant aneurysm likely plays a dominant role. On the other hand, *An* is successful in predicting the overall hemodynamic mode for this case. Realistically, reliable risk assessment requires (at least) a combination of hemodynamics and morphologic parameters [44].

The present study has several limitations. First, this study investigates a single aneurysm anatomy with a specific inflow waveform. Therefore, before generalizing our findings, the results need to be corroborated by similar analysis in multiple geometries and with different physiologically realistic waveforms. Moreover, while our spatial resolution compares favorably with previous experimental studies both *in vitro* and *in vivo*, it might still not be fine enough to accurately resolve the thin boundary layer in the near wall region, which is needed to calculate wall shear stress with high accuracy. Furthermore, although the phase-resolved measurements provide detailed information on the fluid mechanics throughout the cardiac cycle, they cannot capture instantaneous (therefore inherently transient and possibly acyclic) changes in flow features, which may be important at the considered Reynolds numbers [16, 45, 46, 47]. Another source of approximation compared to *in vivo* dynamics is due to a rigid geometry. This is a widespread computational and experimental practice given the lack of sufficient *in vivo* data on patient-specific vessel wall constitutive properties. Some studies using dynamic angiography coupled with CFD models showed marginal effects of the wall motion on the flow pattern but noticeable change in WSS [48], while others using similar approaches reported no significant effect overall [49]. Still, retrospective clinical studies have shown an association between wall motion and rupture status [50]. Recent CFD investigations incorporating fluid-structure interaction found that the aneurysm volume variation during the cardiac cycle is correlated with the probability of rupture [51, 52]. Finally, we have neglected the non-Newtonian nature of the blood. This approximation is typically correct in relatively large brain vessels although it may not be fully justified at smaller vessel sizes [53]. Several aneurysm studies that incorporated non-Newtonian formulations found Newtonian models to be adequate, see e.g. [54].

This study shows the significant amount of information that can be extracted from the complete volumetric and phase-resolved reconstruction of the flow inside an intracranial aneurysm. Although it was not pursued here, this type of data also lends itself to Lagrangian transport analysis, whose power has been shown in numerous computational works, see e.g. [55]. The potential of 4D flow MRI to elucidate important mechanisms in brain aneurysms was recently demonstrated *in vivo* [56]. Imaging of real patients presents challenges that are easily overcome in phantom studies, allowing for higher accuracy and spatial resolution. However, with continuous improvements associated to advanced MRI sequences (e.g. leading to larger dynamic range, [57]) and ever-increasing magnetic field strength [58], one can envision future *in vivo* studies to reach similar performances. Therefore, development of technological advances through *in vitro* studies has a significant potential to improve non-invasive diagnostics with evident long-term benefits for patients.
